# Skin Diseases and Syphilis

**Published:** 1894-10

**Authors:** 


### SKIN DISEASES AND SYPHILIS.

EDITED BY DR. M. B. HUTCHINS.

NOTE.—The following article is of so much interest, so practical and so in accordance with modern views, that the editor takes the liberty of reproducing it in full.

### THE MANAGEMENT OF ECZEMA. By MALCOLM MORRIS.

In dealing with the subject of eczema I propose to present, in as summary a manner as possible, the general results of my own experience in the treatment of this affection, inviting special attention to the points as to which a comparison of experience seems to be most desirable.

I. The first point which I propose for your consideration is: *Are internal remedies required in eczema? If they are, what are the precise indications which should guide us in the use of them?*

My own view, stated broadly, is that, as a general rule, the less internal medication there is in eczema the better. If a constitutional dyscrasia underlie the skin affection, it must of course be treated on the ordinary principles of medicine; if the neurotic element be pronounced, remedies directed to the restoration of the nervous tone are obviously indicated; if the patient be broken down by want of sleep and the exhaustion consequent upon prolonged suffering, sedatives and general tonics will be required; similarly, if there be disorder of the digestive apparatus, this must be rectified by appropriate measures. Where, however, there are no definite indications of the kind that have been referred to, I am strongly of opinion that internal medication is not only useless, but positively injurious by disordering the digestion. In an ordinary chronic eczema, where the patient's health appears to be unaffected, and there is no reason to suspect constitutional dyscrasia, I trust entirely to local treatment. Where internal remedies are given, the selection of the particular drugs employed must be governed by certain definite indications. When the lesions are acutely inflammatory in character, I find antimony most valuable. I begin by giving *m* x to xiij of the vinum antimoniale, repeating the dose in an hour, and, if need be, again two hours later. The interval between the administrations is gradually increased, the dose being at the same time reduced till one of *m* vj is reached. This should be given thrice in the twenty-four hours until a distinct subsidence in the intensity of the inflammation becomes manifest. The indication for antimony is the presence of arterial tension; on the other hand, depression is a positive contraindication of this drug. I am aware that a freer use of antimony has been recommended by some practitioners, but my own experience has not led me to see any necessity for larger doses than those which have been mentioned. If a neurotic element is clearly present in the case, sedatives and nerve tonics must be combined with local treatment. In such cases the sheet anchor is opium, under the influence of which the patient should be kept almost continuously until the nerve storm subsides. In such cases I have often found it of the greatest advantage to begin the treatment with a full dose of calomel, and afterwards the constipating effect of the opium should be counteracted by the administration of a saline aperient, such as Carlsbad salts, Friedrichshall, etc., every morning. If opium disagrees, some other sedative, such as sulphonal, must be substituted. The indication for the use of sedatives is great nervous excitement, accompanied by sleeplessness.

If, on the other hand, the neurotic element shows itself in the form of depression, nerve tonics are indicated; of these the most useful on the whole in my hands has been quinine. This may advantageously be combined with opium. If there be much discharge, belladonna may usefully be combined with the quinine. Phosphorus is another drug of great value in counteracting nervous depression in these cases, and strychnine also often does good. Arsenic, though undoubtedly useful in some cases, is far from being a specific in eczema, and if injudiciously used will do harm instead of good. The indication for its use is deficiency of nerve force combined with absence of acute inflammation in the lesions. If the eruption be of an actively inflammatory type, arsenic is positively contraindicated. If the disease process shows a marked tendency to frequent exacerbations, ergotin may be useful by the control which it exercises over the vaso-motor apparatus.

Malnutrition, weakness, and anemia are indications for cod-liver oil, and general tonic treatment on ordinary principles. Iron, however, is contraindicated by the presence of acute inflammation; in such cases it adds fuel to the flame. Of course, if any definite source of peripheral irritation is discovered, it must, if possible, be removed. In women, menstrual derangement or uterine diseases must be remedied, and hysteria or the disturbances incident to the climacteric period must be combated by such remedies as musk, valerian, etc.

II. The second point which seems to me specially to call for discussion is the *influence of diet in eczema.* I may frankly state that my own view is that diet has no influence at all except indirectly. Thus, if a constitutional condition, such as gout or diabetes, underlie the eczematous process, of course the dietetic restrictions indicated in the circumstances are called for. Again, if the lesions are of an acutely inflammatory type, the diet should be imited in quantity, and bland and non-stimulating in quality. Apart from these indications, I have seen no reason to think that restrictions of diet had any good effect; on the contrary, I have seen a "lowering" regimen do positive harm by weakening the patient. The true principle in the management of eczema, as far as diet is concerned, is to let the patient take such food as he finds most suitable to his digestive powers. Anything, however, which disorders the gastro-intestinal tract, or which causes acidity, insomnia, flatulence, palpitation, or vaso-motor disturbance, must be avoided. The same principle applies to drinks; but here, it is almost needless to add, strict moderation in quantity is necessary. It is only in acute forms that beer is contraindicated, as are all other beverages likely to cause flushing. I place no restrictions on my patients in the use of tea or coffee unless these beverages are contraindicated by definite ill-effects on the digestion or nervous system. In short, as far as diet is concerned in eczema, I

am disposed to say with Bacon, in his essay *On Regimen of Health,* "A man's owne observation, what he findes good of and what he findes hurt of, is the best physicke to preserve health."

III. The third question which I submit for discussion is, *What are the principles on which the local treatment of eczema should be carried out?* It is impossible here to go into the details of the local treatment of eczema of different forms and in different situations; this is the less necessary, as my methods have been fully described elsewhere. Speaking generally, I treat every case as if it were of parasitic origin. Even if micro-organisms are not the exciting agents in the causation of the process in all cases, parasitic irritation always comes into play sooner or later as a secondary factor, and requires to be appropriately dealt with. The objects which I aim at in the local treatment of eczema are, first, to destroy micro-organisms; secondly, to protect the inflamed surface from the air and from further microbic invasion; thirdly, to soothe irritation. The general principles which guide me in the application of local remedies are, first, to temper the strength of the remedy to the tolerance of the patient's skin; and, secondly, to keep the remedy continuously in contact with the affected parts. I therefore begin with a very weak parasiticide, and feel my way towards the use of stronger applications, with the most watchful care not to cause any irritation. The best remedy for local use in dry, chronic eczema, especially of seborrheic origin, in my experience, is sulphur, and next to that I place resorcin. Both these drugs have this special advantage, that they not only destroy the micro-organisms on the surface of the skin, but give rise to exfoliation of the horny layer, and bring away with it the microbes which have penetrated to the deeper parts of the epidermis. I use at first an ointment composed of 10 grains of precipitated sulphur, or the same quantity of resorcin in ℥ j of zinc ointment or some other soothing application; the amount of the antiparasitic agent is increased very gradually. When the inflammation is acute, ichthyol is particularly useful, as it is a vascular sedative as well as a parasiticide. Other antiparasitic remedies which are of service as applications in eczema are salicycle acid, white precipitate, boracic and carbolic acid. I use them with cooling "creams" as vehicles when there is much irritation. For the continuous application of local remedies in limited areas of chronic character, I have found nothing so useful as the plaster mulls devised by Unna, those I find most generally useful being carbolic acid and mercury, and oxide of zinc.

When discharge is profuse I wash the part with a weak solution of boracic acid, afterwards drying it with muslin bags containing starch and powdered boracic acid, or with flour mixed with a small quantity of the same antiseptic. If itching is troublesome, I find weak lotions of carbolic acid or tar most beneficial. For very persistent chronic eczema of the flexures I have frequently had good results from chrysarobin.

I may sum up the principles on which the local treatment of eczema should be conducted as follows: Soothe when the inflammatory process is acute; stimulate when it is chronic; and in either case keep the parts under the continuous influence of antiseptics and parasiticides, of a strength carefully regulated in accordance with the intensity of the disease and the sensitiveness of the skin.

IV. A fourth point on which discussion is likely to be useful is, *How is the tendency to recurrence of eczema in those predisposed to the disease to be overcome? Is there any plan of alterative treatment by which this result can be brought about?*

Change of climate is often productive of good in this direction, especially if accompanied by complete rest of mind as well as body. I know of no special indications in respect to climate except that eczema, being a catarrhal disease, it will be well to avoid climates which are apt to produce catarrh of any kind. A too bracing climate—especially places where the northeast wind holds sway—should be avoided. The same may be said of sea-air. As regards altitude, there is no general rule; as in the case of climate, it is largely a matter of idiosyncrasy. The true principle is to discover what suits the patient. After all, it is the change that is the most powerful alterative. Sea-bathing very often produces a recurrence of the disease, but it sometimes undoubtedly counteracts the tendency to relapse.

As to waters, in estimating their usefulness, the effect of the change of scene and surroundings which a visit to a spa involves must be taken into account, as well as the chemical constitution of the springs. In my experience they are beneficial chiefly, if not so solely, in obstinate chronic cases. In selecting a spring for a particular case we have not much in the way of definite indications to guide us. The alternative influence of spas in eczema is a large subject, which in itself might well occupy the whole time allotted for this discussion. I can only touch on it here in the most cusory way, leaving it to others of large experience in this particular direction to give us the benefit of the knowledge so gained. On the whole, the sulphur springs—especially those of Harrogate and Strathpeffer—are best for external use; but if there be a marked tendency to acute inflammation they are apt to aggravate the disease. The internal use of sulphur waters is particularly indicated in cases in which gout or rheumatism underlies the skin affection. Chalybeate and arsenical waters are useful in cases in which general tonics are indicated. The St. Victor Spring at Royat is one of the richest, both in iron and in arsenic, and is therefore particularly useful for eczema in anemic patients. Of the purely arsenical waters, the most useful in eczema are those of La Bourboule and Levico. The alkaline waters of Vichy and Ems have not seemed to me to be of any particular use in cases of eczema, even in gouty subjects. In many cases the waters of Bath do good. The springs which I have mentioned are those which in my own hands have proved of greater service in cases of eczema.

I may sum up my own conclusions as to the utility of springs in the following propositions:

1. No spring known to me has any specific action on eczema.

2. Such virtue as sulphur waters externally applied possess is due chiefly to the parasiticide action and partly to the temperature at which they are applied.

3. Such virtue as sulphur, chalybeate, or arsenical waters possess when taken internally is due to their action in quickening the processes of metabolism, increasing the number of red corpuscles in the blood, and giving tone to the nervous system.—*From British Journal of Dermatology, September,* 1894.

